# COVID-19: Is it the black death of the 21st century?

**DOI:** 10.34172/hpp.2020.27

**Published:** 2020-07-12

**Authors:** Ali Shamekh, Ata Mahmoodpoor, Sarvin Sanaie

**Affiliations:** ^1^Student Research Committee, Tabriz University of Medical Sciences, Tabriz, Iran; ^2^Anesthesiology and Critical Care Department, School of Medicine, Tabriz University of Medical Sciences, Tabriz, Iran; ^3^Neurosciences Research Center, Aging Research institute, Tabriz University of Medical Sciences, Tabriz, Iran

## Dear Editor,

“*From black death to corona virus: a brief history of quarantines”; “Iran’s coronavirus outbreak is bizarrely reminiscent of the black death”; “Learn from history: black death killed millions of people”; “Black death offers lessons for dealing with covid-19 outbreak”* .

This is just a glimpse at the headlines of some famous papers regarding the outbreak of the novel coronavirus disease of 2019 (COVID-19) highlighting the importance of this crisis as a global warning. On January 31, 2020, the WHO announced the outbreak of COVID-19 as a public health emergency worldwide.^1^ High virulence of the pathogen, its rapid transmissibility, and the potential for environmental contamination are important characteristics of COVID-19, reaffirming the need for special attention. During this pandemic, serious considerations have been given to the high-level quarantine in most countries battling with COVID-19. Many outbreaks have occurred throughout human history, but there is only one outbreak with the first intense quarantine, the plague.^2^ As it’s said, “history repeats itself”. Like COVID-19, the Black Death was an indiscernible, inconspicuous, and mischievous disease; it evolved from the East, spread among the population in cities and towns, and even made its way to different countries through international trade. The history of quarantine dates back to the time of the Black Death (Plague) when medicine was incapable to fight the disease. Avoiding contact with contaminated objects and infected subjects was the only way to evade the disease. Therefore, some regions banned foreigners from entering their province at that time.^3^ In today’s modern world, the long-lived system of quarantine is still a potent part of the public health reaction to newly emerging infectious diseases as well as reemerging ones. In the “Fact Sheets” provided by the CDC, it is stated that “*Quarantine is medically very effective in protecting the public from disease* ”.^4^ Quarantine has been the keystone of coordinated responses to communicable disease epidemics and an organized disease-control strategy. There are other terms such as “home quarantine,” “work quarantine” “self-quarantine” and even “rolling quarantine.” A comprehensive public education on the risk-reducing aspects of quarantine can attract peoples’ interest in the benefits of this method. Quarantines, even though controversial, come at high costs, and have been viewed and implied with suspicion; they are historically proven to delay and decrease the spread of various outbreaks. We should understand different types of quarantines, their combined benefits, the best timing for their implementation and their cost effectiveness. Currently, traditional methods and new techniques merge to form a practical approach to manage disease outbreaks. Since the science of medicine and technology have rapidly improved, treatment approaches of physicians have been directed toward personalized medicine. However, this approach is not achievable during pandemics and mass scenario has to be used for COVID-19 as experienced in the case of the plague. A recently published Cochrane systematic review about the effects of quarantine on the distribution of COVID-19 showed that the combination of quarantine with other preventive and controlling measures had a significant effect on the reduction of transmissions and deaths, and was most effective when applied earlier in the course of disease.^5^ It seems that decisions regarding the selection different types of quarantine by policy makers should be performed based on the cultural and economic properties of each region. Moreover, continuous monitoring of the disease distribution should be locally performed in order to maintain the best measures specifically tailored for every particular region.

**Figure 1:**
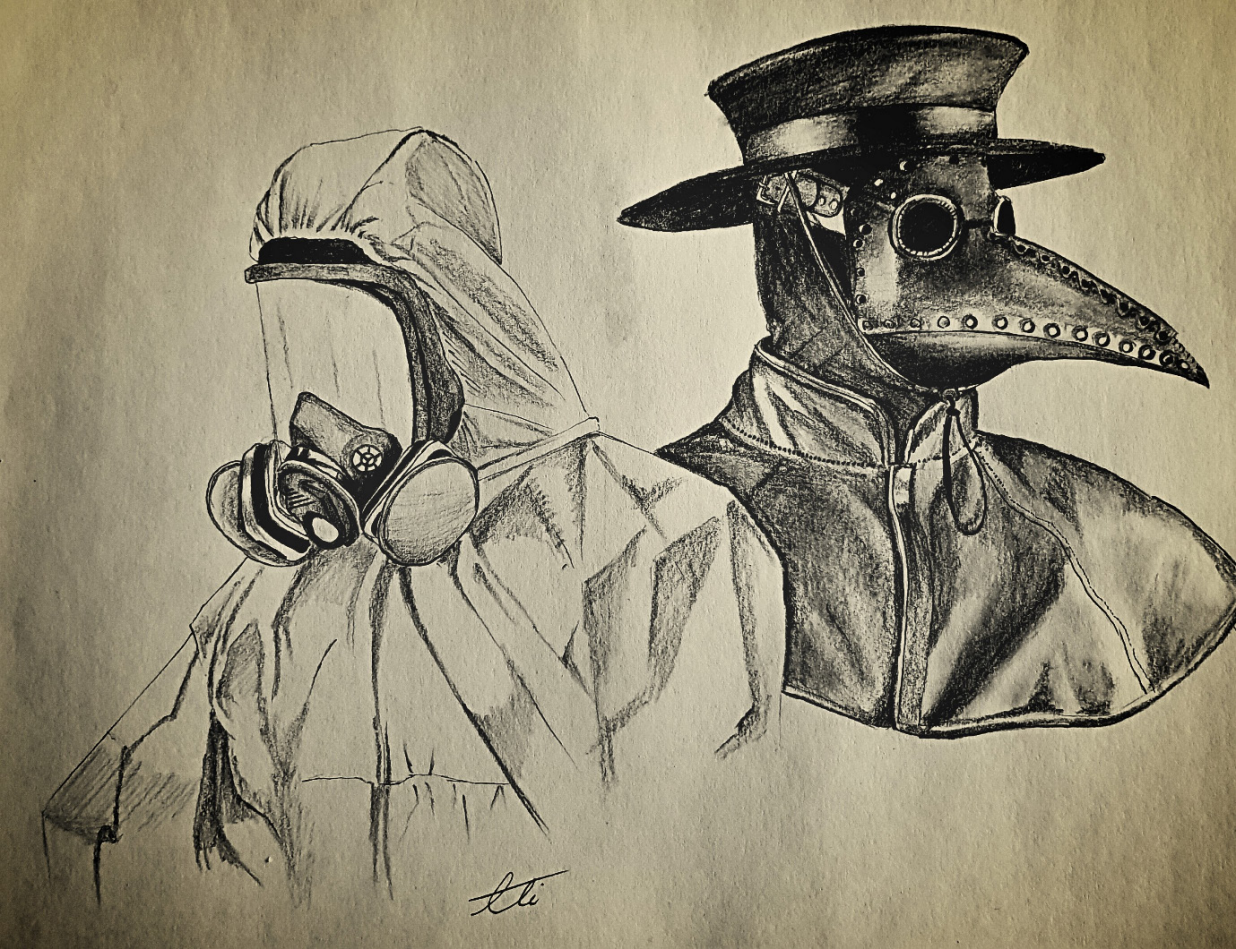
Repetition of history; the rebirth of a new plague.

Another important issue is the safety of healthcare providers that should be highly taken into consideration. Precautions to be implemented by healthcare workers who care for patients with COVID-19 include an appropriate use of personal protective equipment (PPE); this involves selecting the proper PPE and being trained about the correct ways to equipment, removal, and disposal of it (Figure 1).^6^ One of the most iconic PPEs in medicine was used by plague doctors. Their costume consisted of a long overcoat and a beak-shaped mask, regularly filled with aromatic substances, along with a broad-brimmed hat, boots, gloves, and an outer covering clothing (Figure 1). As nowadays the approach to patients with plague has totally been changed and there is no need for such costumes, it is expected that the management situation of COVID-19 patients will also change by a better understanding of the pathogenesis and the structure of the virus; this change may also include some alternations in PPE especially in the masks. The self-protection for health care workers is so important that they can become a potential patient themselves and an involuntary coronavirus spreader while being a caregiver who treats the patients. In spite of the current emphasis on the use of PPE to control the virus transmission, the compliance of healthcare workers is still as not as expected.^7^ The reasons behind this fact may be as the followings: low level of support from the managers or lack of access to equipment, cultural problems, lack of training, not trusting the quality of available PPE, and not having enough desire to deliver a good patient care.

The estimation of future incidence of COVID-19 seems impossible, as several thousands of new cases and dozens of losses are preserved every day in an unpredictable way. The administrations must perceive and imply precise safety agendas, and conclude how to enhance and enforce the current countrywide strategies against COVID-19 pandemic as soon as possible. We believe that an effective defensive mechanism will be found, but it is not yet known when and what this will happen. The most important challenges are timing and choosing a strategy that will not necessarily eradicate the coronavirus, but rather will limit the disease to an endemic. Finally, as the case of plague, we hope that this acute situation with its high incidence and death rates, will change its course to a controlled illness with much lower morbidity and mortality.

## Ethical approval

Not applicable.

## Competing interests

The authors declare that they have no competing interests.

## Authors’ contributions

AS and SS equally contributed to the conception and design of this letter; SS and AM drafted the manuscript. AS painted the figure. AS and AM critically revised the manuscript. All authors read and approved the final manuscript.
